# Primary FSGS is not associated with worse kidney outcome compared with other FSGS subtypes

**DOI:** 10.1093/ckj/sfaf060

**Published:** 2025-02-21

**Authors:** Dries Deleersnijder, Evert Cleenders, Maarten Coemans, Amélie Dendooven, Priyanka Koshy, Kathleen Claes, Katrien De Vusser, Björn K Meijers, Ben Sprangers, Steven Van Laecke, Amaryllis H Van Craenenbroeck

**Affiliations:** Nephrology and Renal Transplantation Research Group, Department of Microbiology, Immunology and Transplantation, KU Leuven, Leuven, Belgium; Nephrology and Renal Transplantation Research Group, Department of Microbiology, Immunology and Transplantation, KU Leuven, Leuven, Belgium; Nephrology and Renal Transplantation Research Group, Department of Microbiology, Immunology and Transplantation, KU Leuven, Leuven, Belgium; Division of Pathology, University Hospital Ghent, Ghent, Belgium; Laboratory of Experimental Medicine and Pediatrics, University of Antwerp, Wilrijk, Belgium; Nephrology and Renal Transplantation Research Group, Department of Microbiology, Immunology and Transplantation, KU Leuven, Leuven, Belgium; Department of Pathology, University Hospitals Leuven, Leuven, Belgium; Nephrology and Renal Transplantation Research Group, Department of Microbiology, Immunology and Transplantation, KU Leuven, Leuven, Belgium; Division of Nephrology, University Hospitals Leuven, Leuven, Belgium; Nephrology and Renal Transplantation Research Group, Department of Microbiology, Immunology and Transplantation, KU Leuven, Leuven, Belgium; Division of Nephrology, University Hospitals Leuven, Leuven, Belgium; Nephrology and Renal Transplantation Research Group, Department of Microbiology, Immunology and Transplantation, KU Leuven, Leuven, Belgium; Division of Nephrology, University Hospitals Leuven, Leuven, Belgium; Department of Nephrology, Ziekenhuis Oost-Limburg, Genk, Belgium; Department of Immunology and Infection, Biomedical Research Institute, UHasselt, Diepenbeek, Belgium; Renal Division, Department of Internal Medicine, Ghent University Hospital, Ghent, Belgium; Nephrology and Renal Transplantation Research Group, Department of Microbiology, Immunology and Transplantation, KU Leuven, Leuven, Belgium; Division of Nephrology, University Hospitals Leuven, Leuven, Belgium

**Keywords:** eGFR slope, epidemiology, focal segmental glomerulosclerosis, FSGS, prognosis

## Abstract

**Background:**

Studies that compare kidney outcomes across patients with different forms of focal segmental glomerulosclerosis (FSGS) are lacking.

**Methods:**

This retrospective study compared annual estimated glomerular filtration rate (eGFR) slope and kidney failure rate (eGFR <15 mL/min/1.73 m^2^ or initiation of kidney replacement therapy) across patients with biopsy-proven primary, maladaptive, genetic and undetermined FSGS. Patients were included from two Belgian tertiary referral hospitals, from 2010 until 2022. Associations between covariates and kidney failure were estimated using Cox and Fine and Gray models. eGFR slopes were estimated using linear mixed-effects models.

**Results:**

Eighty-two patients were subdivided into primary (28.1%), maladaptive (40.2%), genetic (14.6%) and undetermined FSGS (17.1%) groups. Kidney failure occurred in 22 patients (26.8%). Primary FSGS patients exhibited higher baseline eGFR and less chronic changes on biopsy. The annual eGFR slope was –2.5 mL/min in primary, –2.5 mL/min in maladaptive, –4.6 mL/min in genetic and –4.4 mL/min in undetermined FSGS. Female sex was associated with a lower kidney failure rate and higher eGFR slope. Higher proteinuria at biopsy was associated with a higher kidney failure rate, lower eGFR slope and a higher mortality rate. Global sclerosis on kidney biopsy was associated with lower baseline eGFR, while a higher percentage of segmental sclerosis rather associated with more rapid eGFR decline [–1.5 mL/min/year per 10% increase, 95% confidence interval (–2.2, –0.7)].

**Conclusions:**

Patients with primary FSGS were biopsied earlier in their disease course and exhibited surprisingly good kidney outcome. Overall, sex, baseline eGFR, proteinuria and the degree of focal and global glomerulosclerosis play a more important role in estimating the prognosis of patients with FSGS than merely the FSGS etiology.

KEY LEARNING POINTS **What was known:**

- Focal segmental glomerulosclerosis (FSGS) lesions are caused by a wide range of clinical conditions, subdivided into primary, secondary and genetic etiologies. However, previous studies generally focused on primary FSGS or made no distinction at all regarding the underlying etiology.
- Secondary FSGS caused by glomerular hyperfiltration, also known as maladaptive FSGS, is thought to have a better kidney outcome, compared with primary FSGS. However, this dogma has been recently challenged by a recent retrospective study that found a more rapid estimated glomerular filtration rate (eGFR) decline in secondary FSGS patients.

**This study adds:**

- This study aimed to, first, better classify FSGS patients across their presumed underlying etiology and, second, study difference in kidney outcome across etiologies, by assessing both annual eGFR slope and kidney failure.
- Patients with primary FSGS were biopsied earlier in their disease course and did not show accelerated kidney function decline, nor higher rates of kidney failure. However, patients with genetic FSGS did show a more rapid eGFR decline.
- Male sex, lower baseline eGFR, higher baseline proteinuria and a higher degree of glomerulosclerosis were associated with adverse outcomes in FSGS, regardless of the FSGS etiology. A higher percentage of segmental sclerosis was associated with more rapid kidney function decline, likely reflecting more aggressive disease.

**Potential impact:**

- Patients with genetic FSGS may exhibit rapid kidney function decline, warranting close nephrological follow-up.
- A high percentage of glomeruli affected by FSGS lesions may be considered as a new parameter to be included in risk stratification in patients with FSGS, regardless of the underlying FSGS etiology.
- In approximately one out of six patients, the precise cause of the FSGS lesions remained unknown, highlighting the need for fundamental research into novel serum diagnostic biomarkers in primary FSGS.

## INTRODUCTION

Focal segmental glomerulosclerosis (FSGS) is defined by the microscopical appearance of glomerulosclerosis in segments of some (“focal”) glomeruli [1–5]. FSGS lesions result from critical injury to podocytes, leading to an impaired filtration barrier, proteinuria and kidney function decline. Podocytes respond to injury through the loss of their interdigitating foot process pattern, in a process called foot process effacement (FPE), mediated by remodeling of their actin cytoskeleton [5]. During FPE, filtration slits start closing, and the interdigitating feet are transformed into flat plate-like cytoplasmic sheets that cover the glomerular basement membrane (GBM) [5]. If such injury is prolonged, podocytes finally detach, leaving regions of the GBM uncovered, resulting in glomerulosclerosis [5, 6]. Historically, FSGS was defined as a separate disease entity, while it is now recognized that FSGS lesions occur in a wide range of clinical conditions that are all characterized by severe podocyte injury, also referred to as podocytopathies [5, 7]. Podocytopathies are currently subdivided into primary, secondary and genetic forms, each with different outcomes and therapeutic options [4].

Primary FSGS is caused by one or several circulating permeability factors that cause podocyte injury and is typically treated with immunosuppressive therapy [4, 5]. Pathogenic circulating anti-nephrin auto-antibodies have recently been implicated in disease pathophysiology, as they have been detected in 9% of patients with primary FSGS [8], and in all patients of a small cohort with post-transplant recurrence of FSGS [9]. Secondary FSGS is further subdivided based on the underlying cause. Most often, FSGS lesions arise secondary to glomerular hypertension, also referred to as maladaptive FSGS, in which there is a mismatch between glomerular load and glomerular capacity [10]. Maladaptive FSGS can occur in patients with reduced kidney mass (e.g. low nephron endowment at birth, reflux nephropathy), but more often in patients with a normal number of nephrons that are subjected to various causes of hyperfiltration (e.g. obesity, obstructive sleep apnea) [10]. Maladaptive FSGS lesions also occur in patients with other primary glomerular disease: in these cases, FSGS lesions are superimposed on the primary kidney disorder, and rather a sign of chronic damage caused by the primary etiology. The treatment of maladaptive FSGS is aimed at reducing hyperfiltration with renin–angiotensin system blockade. Finally, the genetic forms of FSGS result from pathogenic variants in genes that encode for podocyte proteins or collagen type IV, and are typically resistant to therapy [4, 5, 11]. In adult-onset genetic FSGS, causative genetic variants are most often detected in collagen genes (in about 50%–60% of cases) [11, 12].

Epidemiological differences across FSGS etiologies have been inadequately studied: previous observational studies generally focused on primary FSGS [13, 14], or made no distinction at all regarding the underlying etiology of FSGS lesions. Based on the results from two older studies that compared primary with obesity-related secondary FSGS, it was assumed that patients with secondary FSGS exhibit better kidney outcomes [1, 2]. Recently, this dogma has been challenged by a retrospective study from Germany, that found that secondary (mostly maladaptive) FSGS patients show a more rapid estimated glomerular filtration rate (eGFR) decline compared with patients with primary FSGS [3].

To further investigate this discrepancy, we conducted a retrospective study of biopsy-proven FSGS patients in two tertiary referral hospitals in Belgium, with two aims. First, we aimed to better classify patients with FSGS according to the underlying etiology, by applying strict diagnostic criteria [5, 15]. Next, we aimed to compare outcomes in different FSGS subgroups and identify potential predictors for poor kidney outcome.

## MATERIALS AND METHODS

### Ethics

This study was approved by the ethical committees of the hospitals UZ Leuven and UZ Ghent (study reference S66295).

### Study design and population

Adult patients with a biopsy-confirmed diagnosis of FSGS in UZ Leuven and UZ Ghent (biopsied in time interval 2010 to 2022) were eligible for inclusion. In case of repeated biopsies, only the first biopsy was included. Additionally, in two patients, the first biopsy showed a diagnosis of minimal change disease (MCD), while the repeat biopsy indicated FSGS lesions (likely due to sampling error in the first biopsy): in these cases, the first MCD biopsy (prior to start of immunosuppressive treatment) was included as the baseline timepoint. Patients with no immunofluorescence or immunohistochemistry evaluation on kidney biopsy were excluded. Additionally, patients in which FSGS lesions appeared secondary to another primary glomerular disease, drugs or viral infections were also excluded. Data missingness was low (Supplementary data, Table S1).

### Clinicopathologic characterization of FSGS etiology

Patients were stratified according to their presumed underlying FSGS etiology (primary, maladaptive, genetic and undetermined FSGS), using all diagnostic criteria outlined in Table 1. Nephrotic syndrome was defined as the presence of serum albumin <35 g/L (using bromocresol green assay) AND urine protein–creatinine ratio (UPCR) ≥3.5 g/g or proteinuria ≥3.5 g on 24 h urinary collection, with or without edema, in a period of 3 months before or 3 months after kidney biopsy. We included the Mayo Clinic Chronicity Score (MCCS) to uniformly score chronic changes in kidney biopsies, which is categorically classified into minimal (MCCS 0–1), mild (MCCS 2–4), moderate (MCCS 5–7) and severe (MCCS 8–10) chronic changes (Supplementary data, Table S2) [16]. FSGS and global sclerosis lesions were included as a percentage of total glomeruli on light microscopy.

**Table 1:** FSGS etiologies and diagnostic criteria used in stratification.

		Maladaptive FSGS			
	Primary FSGS	With identified cause	Without identified cause	Genetic FSGS	Undetermined FSGS
Nephrotic syndrome	Present	Absent	Absent	Present or absent	Patients not meeting the criteria of the other groups
Electron microscopy^a^	≥80% podocyte FPE	<80% podocyte FPE	<80% podocyte FPE	Any FPE	
Secondary causes	Not present	Maladaptive^c^ (drugs and viral infections excluded)	Not present (drugs and viral infections excluded)	Not present	
Genetics^b^	Negative or not performed	Negative or not performed	Negative or not performed	(Likely) pathogenic variant	

a Data on FPE on electron microscopy were available in 15 primary FSGS samples (65.2%), 14 maladaptive FSGS samples (42.4%), 6 genetic FSGS samples (50.0%) and 6 undetermined FSGS samples (42.9%).

b Presence of pathogenic variant or likely pathogenic variant on genetic testing with “proteinuria” panel.

^c^Maladaptive causes were defined as: obesity (BMI ≥30 kg/m^2^), history of obstructive sleep apnea, history of diabetes mellitus, history of macrovascular disease (composite of coronary heart disease and/or cerebrovascular disease and/or peripheral artery disease) and reduced nephron number (reflux nephropathy, nephrectomy, unilateral renal dysplasia or agenesis, oligomeganephronia, low birth weight).

### Statistical analysis

#### Baseline characteristics

All analyses were performed in R (https://www.R-project.org/). Continuous variables were described by median and interquartile range (IQR), categorical variables by frequencies and percentages. Differences between continuous variables that showed normal distribution and equal variance were assessed using one-way analysis of variance, and Tukey's tests for post-hoc pairwise comparisons. For non-normally distributed continuous variables, Kruskal–Wallis tests and post-hoc pairwise Bonferroni-corrected Dunn tests were used. Differences between categorical variables were assessed using Fisher's exact tests and Bonferroni-corrected pairwise Fisher's exact tests. (Adjusted) *P*-values of <.05 were considered significant.

#### Outcome parameters

Kidney failure and all-cause mortality were evaluated from January 2010 to December 2022. Kidney failure was a composite endpoint, including eGFR CKD-EPI (Chronic Kidney Disease Epidemiology Collaboration) <15 mL/min/1.73 m^2^ (lasting for ≥3 months), kidney transplantation or initiation of chronic dialysis, whichever occurred first. For kidney failure, “death before kidney failure” was a competing event, which occurred in eight patients; patients in which no kidney failure occurred were right-censored at the date of the last available eGFR measurement. For all-cause mortality, no competing events were present. Patients who survived throughout the study period were right-censored at the date of the last medical follow-up.

#### Cumulative incidence analysis

The Aalen–Johansen estimator was used to plot the cumulative incidence of kidney failure over time, treating death as a competing event [17]. For cumulative incidence estimation of death, the Kaplan–Meier estimator was used. Comparisons between groups were performed with the Gray's test and the log-rank test, respectively.

#### Cause-specific (Cox) and subdistribution (Fine and Gray) hazard approaches

Univariable and multivariable Cox proportional hazards regression was used to model the etiological relation between covariates and (i) kidney failure (censored for competing event “death before kidney failure”), (ii) death before kidney failure (censored for competing event kidney failure) and (iii) any death [17]. Cause-specific hazard ratios (HR) were presented with 95% confidence interval (CI). Univariable and multivariable (competing risks) Fine and Gray models were additionally used to model the relation between covariates and the risk of kidney failure [17]. Subdistribution hazard ratios (sHR) were presented with 95% CI. *P*-values of <.05 were considered significant.

#### Linear mixed-effects models for eGFR slope

Univariable and multivariable linear mixed-effects models were used to estimate the annual eGFR slope, including random intercepts and random slopes. Additional details are provided in the Supplementary Methods. In primary FSGS, the eGFR fluctuated in the first 3 months after the biopsy and was often not indicative for the long-term eGFR slope. Therefore, the baseline timepoint for intercept and slope analysis was set at 3 months after kidney biopsy for all FSGS subgroups. Estimates (β) were presented with 95% CI. *P*-values of <.05 were considered significant.

## RESULTS

### Baseline demographics and clinicopathologic characteristics

**Figure 1:**
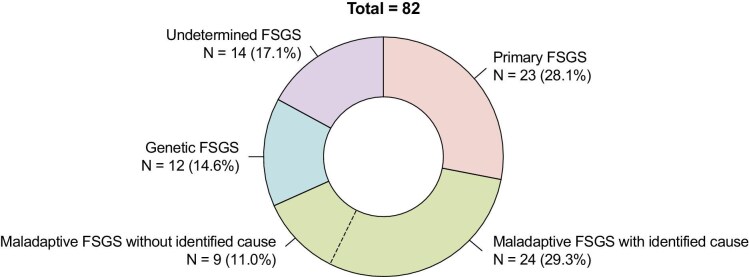
Subdivision of FSGS patients across etiologies.

A total of 82 patients were subdivided into primary FSGS (*N* = 23, 28.1%), maladaptive FSGS with identified cause (*N* = 24, 29.3%), maladaptive FSGS without identified cause (*N* = 9, 11.0%), genetic FSGS (*N* = 12, 14.6%) and FSGS of undetermined etiology (*N* = 14, 17.1%) (Fig. 1). Details on patients with FSGS of undetermined etiology are shown in Supplementary data, Table S3. For comparative analyses, both maladaptive FSGS groups were pooled together (*N* = 33, 40.2%). Baseline characteristics are shown in Table 2. Age, sex and body mass index (BMI) were not significantly different between groups. The percentage of former or active smokers was high in all groups (overall 59.8%). Primary FSGS patients were most often nephrotic at biopsy (87.0%), followed by undetermined (58.3%), genetic (9.1%) and maladaptive (0.0%) FSGS (*P* < .001). Proteinuria (UPCR) around diagnosis was greatest in primary FSGS [7.9 g/g (IQR 5.8–9.5)], followed by undetermined [4.6 (IQR 3.4–6.6)], maladaptive [2.1 (IQR 0.7–3.0)] and genetic [2.1 (IQR 1.4–3.4)] FSGS (*P* < .001). eGFR at biopsy was lowest in undetermined FSGS [35.0 mL/min/1.73 m^2^ (IQR 29.0–69.0)] and highest in primary FSGS [61.0 mL/min/1.73 m^2^ (IQR 33.5–90.0)], but differences were not statistically significant (*P* = .450).

**Table 2:** Baseline characteristics.

	Primary FSGS (*N* = 23), median (IQR) or *N* (%)		Maladaptive FSGS (*N* = 33), median (IQR) or *N* (%)		Genetic FSGS (*N* = 12), median (IQR) or *N* (%)		Undetermined FSGS (*N* = 14), median (IQR) or *N* (%)		*P*-value
Demographics and comorbidities									
Age, at biopsy, years	46.1	(40.7–68.4)	58.2	(44.8–68.7)	45.8	(39.1–49.4)	55.3	(43.9–60.6)	.179
Sex, male	14	(60.9)	25	(75.8)	5	(41.7)	7	(50.0)	.126
Ethnicity, White	21	(91.3)	28	(84.9)	12	(100.0)	13	(92.9)	.592
BMI, at biopsy (kg/m^2^)	27.1	(25.8–29.9)	26.7	(22.5–31.9)	25.2	(23.8–28.1)	27.1	(26.1–33.1)	.254
BMI, 1 month after biopsy (kg/m^2^)	25.4	(23.1–27.4)	29.2	(22.5–33.3)	25.1	(24.1–27.6)	26.5	(25.9–34.5)	.168
OSAS, at biopsy	2	(8.7)	5	(15.2)	1	(8.3)	1	(7.1)	.884
Hypertension, at biopsy	11	(47.8)	24	(72.7)	10	(83.3)	12	(85.7)	.057
Current/former smoker, at biopsy	14	(60.9)	21	(63.6)	6	(50.0)	8	(57.1)	.883
Diabetes mellitus, at biopsy	4	(17.4)	7	(21.2)	0	(0.0)	3	(21.4)	.399
Clinical and biochemical characteristics									
RAAS inhibitor use, at biopsy	11	(47.8)	18	(54.5)	9	(75.0)	9	(64.3)	.430
Nephrotic syndrome, at biopsy	20	(87.0)	0.0	(0.0)	1	(9.1)	7	(58.3)	**<.001** **^a^**,**^b^**,**^c^**
Edema, at biopsy	22	(95.7)	2	(6.5)	1	(8.3)	7	(50.0)	**<.001** **^a^**,**^b^**,**^c^**,**^e^**
Hematuria, at biopsy	18	(81.8)	10	(32.3)	9	(81.8)	10	(76.9)	**<.001** **^b^**,**^c^**,**^f^**
UPCR, highest^g^	7.9	(5.8–9.5)	2.1	(0.7–3.0)	2.1	(1.4–3.4)	4.6	(3.4–6.6)	**<.001** **^a^**,**^b^**,**^c^**
UPCR, at biopsy	6.2	(3.3–8.4)	1.2	(0.2–1.8)	1.9	(1.2–2.8)	3.8	(3.0–6.2)	**<.001** **^a^**,**^b^**,**^c^**
sAlb, at biopsy (g/L)	23.5	(19.2–28.3)	41.2	(40.6–43.0)	41.3	(38.0–43.2)	32.3	(27.4–35.1)	**<.001** **^a^**,**^b^**,**^c^**,**^d^**
sCr, at biopsy (mg/dL)	1.2	(1.0–2.0)	1.7	(1.1–2.2)	1.5	(0.8–2.4)	1.7	(1.1–2.4)	.626
eGFR, at biopsy (mL/min/1.73 m^2^)	61.0	(33.5–90.0)	42.0	(31.0–66.0)	56.5	(29.8–84.0)	35.0	(29.0–69.0)	.450
Histopathologic features									
Percentage of glomeruli with FSGS lesions on LM	10.5	(6.8–18.2)	9.3	(5.5–20.0)	13.4	(7.2–20.0)	13.9	(7.4–27.1)	.756
Percentage of glomeruli with global sclerosis on LM	0.0	(0.0–8.7)	18.2	(8.3–42.9)	25.0	(5.6–30.8)	20.8	(11.8–52.5)	**<.001** **^a^**,**^b^**,**^e^**
GS	1.0	(0.0–1.0)	2.0	(1–2.0)	2.0	(1.0–2.0)	2.0	(1.0–3.0)	**.002** **^b^**,**^e^**
IF	0.0	(0.0–1.0)	1.0	(1.0–2.0)	2.0	(0.0–2.0)	2.0	(0.3–3.0)	.057
TA	0.0	(0.0–1.0)	1.0	(0.0–2.0)	2.0	(0.0–2.0)	2.0	(0.3–2.8)	.060
CV	0.0	(0.0–1.0)	0.0	(0.0–1.0)	1.0	(0.0–1.0)	0.0	(0.0–1.0)	.753
MCCS	2.0	(1.0–4.8)	5.0	(3.0–7.0)	6.0	(2.0–7.0)	6.5	(2.3–8.8)	**.016** **^b^**,**^e^**
EM performed	17	(73.9)	20	(60.6)	10	(83.3)	9	(64.3)	.496
EM showing open glomeruli^h^	15	(88.2)	15	(75.0)	7	(70.0)	8	(88.9)	.550
FPE ≥80%^i^	15	(100.0)	0	(0.0)	0	(0.0)	3	(50.0)	**<.001** **^a^**,**^b^**
Genetics									
Genetics performed	7	(30.4)	9	(27.3)	12	(100.0)	4	(28.6)	**<.001** **^a^**,**^d^**,**^f^**
Initiated treatment(s) and outcomes									
Corticosteroid use	20	(87.0)	0	(0.0)	0	(0.0)	5	(35.7)	**<.001** **^a^**,**^b^**,**^c^**,**^e^**
Tacrolimus use	7	(30.4)	0	(0.0)	0	(0.0)	2	(14.3)	**.001** **^b^**
Ciclosporin use	4	(17.4)	0	(0.0)	0	(0.0)	1	(7.1)	**.039**
Rituximab use	2	(8.7)	0	(0.0)	0	(0.0)	1	(7.1)	.223
Follow-up time KF, months^j^	42.0	(24.9–63.3)	32.3	(22.1–46.2)	37.1	(17.1–70.2)	37.3	(24.5–62.4)	.849
Follow-up time death, months^k^	45.7	(25.5–64.5)	46.1	(27.4–92.2)	71.3	(48.7–80.0)	46.1	(34.4–86.6)	.371
Kidney failure	3	(13.0)	8	(24.2)	5	(41.7)	6	(42.9)	.127
Death	2	(8.7)	5	(15.2)	0	(0.0)	3	(21.4)	.362

a Pairwise testing primary vs genetic FSGS showed adjusted *P*-value <.05.

b Pairwise testing primary vs maladaptive FSGS showed adjusted *P*-value <.05.

c Pairwise testing maladaptive vs undetermined FSGS showed adjusted *P*-value <.05.

d Pairwise testing genetic vs undetermined FSGS showed adjusted *P*-value <.05.

e Pairwise testing primary vs undetermined FSGS showed adjusted *P*-value <.05.

f Pairwise testing maladaptive vs genetic FSGS showed adjusted *P*-value <.05.

g Highest proteinuria in the time interval of 3 months up until biopsy.

h Percentages calculated in cases where EM was performed.

i Percentages calculated in cases where open glomeruli were identified on EM and the degree of FPE was described by the pathologist (15 primary, 14 maladaptive, 6 genetic and 6 undetermined FSGS cases).

j Time from biopsy to kidney failure or censoring (death before kidney failure or last eGFR measurement) in months.

k Time from biopsy to death or censoring (last medical follow-up).

CV, arteriosclerosis (score 0–1, part of total MCCS); EM, electron microscopy; GS, glomerulosclerosis (score 0–3, part of total MCCS); IF, interstitial fibrosis (score 0–3, part of total MCCS); KF, kidney failure; LM, light microscopy; OSAS, obstructive sleep apnea syndrome; RAAS, renin–angiotensin–aldosterone system; sAlb, serum albumin; sCr, serum creatinine; TA, tubular atrophy (score 0–3, part of total MCCS).

*P*-value <.05 shown in bold.

The percentages of glomeruli with FSGS lesions on biopsy were similar across all groups (*P* = .756) (Table 2). In contrast, the percentage of glomeruli with global sclerosis was significantly lower in primary FSGS [0.0% (IQR 0.0–8.7)], compared with maladaptive [18.2% (IQR 8.3–42.9)], genetic [25.0% (IQR 5.6–30.8)] and undetermined FSGS [20.8% (IQR 11.8–52.5)] (*P* < .001). The overall chronicity, summarized by the MCCS, was also significantly lower in primary FSGS [2.0 (IQR 1.0–4.8)] compared with maladaptive [5.0 (IQR 3.0–7.0)], genetic [6.0 (IQR 2.0–7.0)] and undetermined [6.5 (IQR 2.3–8.8)] groups (*P* < .016). Evaluation by electron microscopy (EM) was done in a similar proportion of patients across the groups (ranging from 60.6% to 83.3%) (*P* = .496). In primary FSGS, all biopsies showed diffuse FPE (≥80%), in undetermined FSGS 50% of biopsies showed diffuse FPE, while in maladaptive and genetic FSGS, all biopsies showed <80% FPE (*P* < .001).

### Pathogenic *COL4A* gene variants most often underly genetic FSGS

In all patients with genetic FSGS (*N* = 12), (likely) pathogenic variants were identified in genes known to cause genetic FSGS (Supplementary data, Table S4). In eight patients (66.7%) heterozygous (likely) pathogenic variants in collagen type IV genes (*COL4A3, COL4A5*) were causative. Genetic analysis was less frequently performed in primary (30.4%), maladaptive (27.3%) and undetermined FSGS (28.6%) (*P* < .001), which showed no pathogenic variants in causative genes.

### Use of immunosuppressive treatments across FSGS subgroups

**Figure 2:**
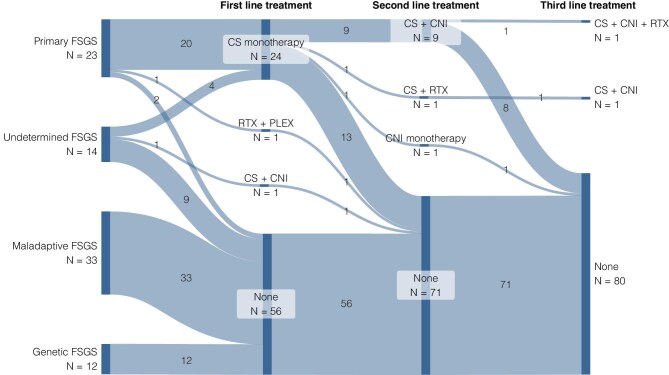
Use of immunosuppressive treatments in FSGS patients. Sankey diagram showing the number of patients with FSGS treated with first-, second- and third-line immunosuppressive treatments. One patient with primary FSGS had a concomitant diagnosis of B-cell non-Hodgkin's lymphoma and was treated with rituximab, bendamustine and plasmapheresis as first-line treatment. Two patients with primary FSGS did not receive any immunosuppressive treatment because they presented with a high degree of chronic damage on kidney biopsy. CNI, calcineurin inhibitor; CS, corticosteroids; PLEX, plasmapheresis; RTX, rituximab.

The use of immunosuppressive treatments across all groups is shown in Fig. 2. Twenty (87.0%) patients with primary FSGS received corticosteroids. None of the patients with maladaptive and genetic FSGS received immunosuppressive treatment. In patients with FSGS of undetermined cause, 5 out of 14 (35.7%) received immunosuppressive treatment during the disease course.

### Cumulative incidence of kidney failure and death does not significantly differ across FSGS subgroups

**Figure 3:**
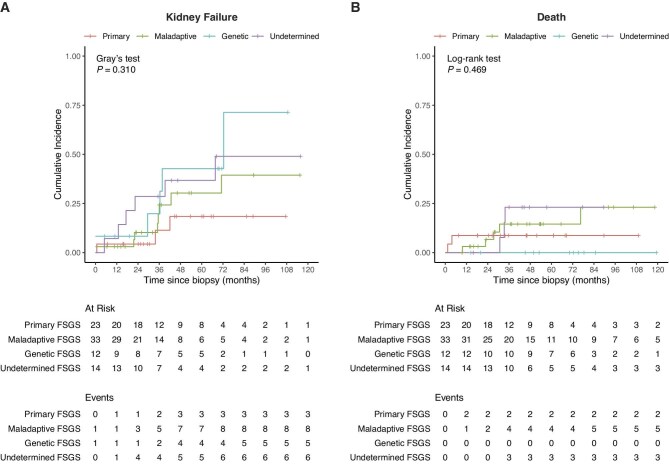
Cumulative incidence of kidney failure and all-cause mortality across FSGS subtypes. (**A**) Aalen–Johansen curves showing the cumulative incidence of kidney failure across FSGS subgroups, accounting for the competing event “death before kidney failure.” Primary FSGS shown in red, maladaptive FSGS in green, genetic FSGS in blue and undetermined FSGS in purple. (**B**) Inverse Kaplan–Meier curves showing the cumulative incidence of any death (either before or after kidney failure), across FSGS subgroups (no competing events present).

Overall, kidney failure occurred in 22 patients (26.8%) over a median follow-up period of 35.4 months (time from biopsy to kidney failure or censoring, IQR 22.2–64.3) and death in 10 patients (12.2%) over a median follow-up period of 46.9 months (time from biopsy to death or censoring, IQR 27.8–78.2) (details on follow-up time in Supplementary data, Table S5). The cumulative incidence of kidney failure and death was not significantly different between the FSGS subgroups (*P* = .310 for kidney failure, *P* = .469 for all-cause mortality) (Fig. 3).

### Higher degree of segmental or global glomerulosclerosis is associated with higher kidney failure rate

**Figure 4:**
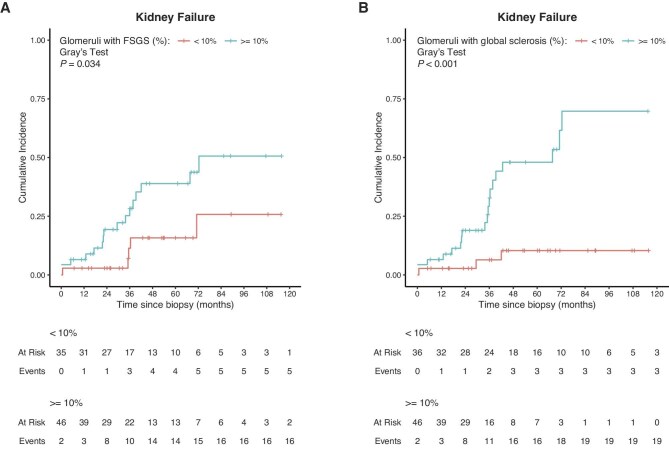
Cumulative incidence of kidney failure according to percentage of focal or global glomerulosclerosis. Aalen–Johansen curves showing the cumulative incidence of kidney failure according to the percentage of glomeruli with FSGS lesions (**A**) or global sclerosis (**B**) on kidney biopsy, accounting for the competing event “death before kidney failure.”

We used uni- and multivariable Cox proportional hazards models to estimate the cause-specific hazard for developing kidney failure for a set of clinicopathologic variables (Table 3). Compared with primary FSGS, maladaptive, genetic and undetermined FSGS were not associated with a significantly different kidney failure rate. In uni- and multivariable analysis, female sex [multivariable, HR 0.198, 95% CI (0.047, 0.836), *P* = 0.028] and higher baseline eGFR [per 10 mL/min/1.73 m^2^; multivariable, HR 0.613, 95% CI (0.388, 0.968), *P* = .036] were associated with a lower kidney failure rate. Proteinuria at baseline was only associated with a higher kidney failure rate in the multivariable analysis [per 1 g/g; HR 1.389, 95% CI (1.074, 1.795), *P* = 0.012]. A higher percentage of glomeruli affected by FSGS [per 10%; multivariable, HR 1.862, 95% CI (1.285, 2.698), *P* = .001] or global sclerosis [per 10%; multivariable, HR 2.201, 95% CI (1.479, 3.275), *P* < .001] were both associated with a significantly higher kidney failure rate. The cumulative incidence of kidney failure was also significantly increased in patients in which ≥10% of glomeruli showed FSGS lesions (*P* = .034, Fig. 4A) or global sclerosis (*P* < .001, Fig. 4B), compared with <10% of glomeruli.

**Table 3:** Cause-specific Cox proportional hazards models for kidney failure.

	Univariable			Multivariable		
Parameter	HR	(95% CI)	*P*-value	HR	(95% CI)	*P*-value
FSGS subgroups						
Primary FSGS (reference)						
Maladaptive FSGS	2.164	(0.572, 8.178)	.255	0.894	(0.052, 15.453)	.939
Genetic FSGS	3.138	(0.747, 13.175)	.118	2.070	(0.086, 49.769)	.654
Undetermined FSGS	3.269	(0.817, 13.084)	.094	1.501	(0.146, 15.392)	.732
Sex						
Male (reference)						
Female	**0.382**	**(0.148, 0.988)**	**.047**	**0.198**	**(0.047, 0.836)**	**.028**
Age, per decade	**1.341**	**(1.005, 1.787)**	**.046**	1.489	(0.862, 2.573)	.154
eGFR at biopsy, per 10 mL/min/1.73 m^2^	**0.636**	**(0.493, 0.820)**	**<.001**	**0.613**	**(0.388, 0.968)**	**.036**
UPCR at biopsy, per 1 g/g	1.087	(0.974, 1.213)	.137	**1.389**	**(1.074, 1.795)**	**.012**
Glomeruli with FSGS (per 10%)	1.230	(0.986, 1.534)	.066	**1.862**	**(1.285, 2.698)**	**.001**
Glomeruli with global sclerosis (per 10%)	**1.677**	**(1.382, 2.034)**	**<.001**	**2.201**	**(1.479, 3.275)**	**<.001**
MCCS (per 1)	**1.631**	**(1.345, 1.978)**	**<.001**			

Univariable and multivariable Cox proportional hazards models estimating the effect of the following clinicopathologic variables on the hazard of kidney failure: FSGS subgroup, sex, age (per decade), eGFR at biopsy (per 10 mL/min/1.73 m^2^), proteinuria at biopsy (UPCR, g/g), the percentage of glomeruli affected by FSGS lesions (per 10%), the percentage of glomeruli affected by global sclerosis (per 10%) and MCCS (per 1 point). Censoring was applied for the competing event “death before kidney failure.” HR is the (cause-specific) hazard ratio. MCCS was not included in the multivariable analysis, as it is colinear with the percentage of glomeruli affected by FSGS and global sclerosis.

Hazard ratios with *P*-value <.05 shown in bold.

To account for patients that die before progressing to kidney failure, we used Fine and Gray models that estimate the

relationship between the covariates and absolute risk of kidney failure (Supplementary data, Table S6). Male sex and higher proteinuria were not significantly associated with a higher risk of kidney failure, due to the higher occurrence of the competing event in these subgroups. Indeed, Cox models that estimated the hazard of “death before kidney failure” showed a trend, yet non-significant, towards higher survival in female patients [multivariable, HR 0.242, 95% CI (0.019, 3.033), *P* = .272] and lower survival in patients with higher baseline proteinuria [multivariable, per 1 g/g, HR 1.724, 95% CI (0.981, 3.029), *P* = .058] (Supplementary data, Table S7). In line with this observation, Cox models that estimated the hazard for any death, either before or after kidney failure, showed a significantly lower survival rate in patients with higher baseline proteinuria [multivariable, per 1 g/g, HR 1.629, 95% CI (1.153, 2.300), *P* = .006] (Supplementary data, Table S8).

### Genetic FSGS, male sex, proteinuria and percentage of FSGS lesions are associated with more rapid kidney function decline

**Figure 5:**
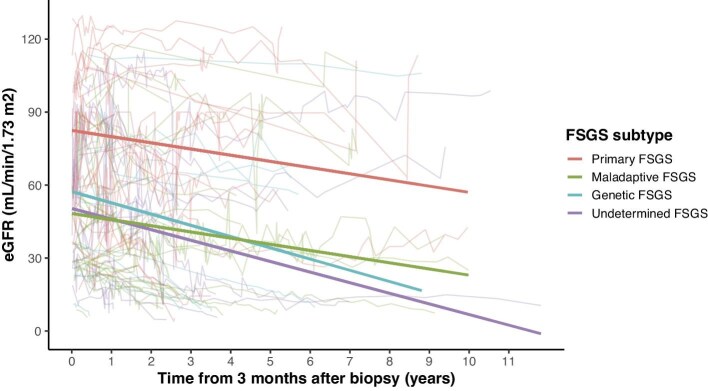
Baseline eGFR and slope in different FSGS subgroups. Univariable linear mixed-effects model estimating eGFR slope across FSGS subgroups. Timepoint 0 was set at 3 months after kidney biopsy. The thin curves depict individual patient observations, thick curves represent the fitted regression lines for the FSGS subgroups.

To estimate the effect of the FSGS subgroups on baseline eGFR and slope, a univariable linear mixed-effects model was applied (Fig. 5, Table 4). Compared with primary FSGS, estimated baseline eGFR was significantly lower in maladaptive FSGS [–34.1 mL/min, 95% CI (–49.7, –18.6), *P* < .001], genetic FSGS [–25.2 mL/min, 95% CI (–45.3, –5.0), *P* = .015] and undetermined FSGS [–32.1 mL/min, 95% CI (–51.6, –12.6), *P* = .002]. The estimated annual eGFR slope was –2.5 mL/min in primary FSGS, –2.5 mL/min in maladaptive FSGS, –4.6 mL/min in genetic FSGS and –4.4 mL/min in undetermined FSGS, but these differences did not reach statistical significance.

**Table 4:** Linear mixed-effects models estimating baseline eGFR and annual eGFR slope.

	Univariable			Multivariable		
	β	95% CI	*P*-value	β	95% CI	*P*-value
**Main effect** ^a^						
(Intercept)	**82.460**	**(70.549, 94.370)**	**<.001**	**87.180**	**(73.472, 100.889)**	**<.001**
FSGS subgroups						
Primary FSGS (reference)						
Maladaptive FSGS	**–34.124**	(**–49.682, –18.567)**	**<.001**	**–22.057**	(**–39.219, –4.895)**	**.013**
Genetic FSGS	**–25.166**	(**–45.341, –4.992)**	**.015**	**–21.365**	(**–42.519, –0.212)**	**.048**
Undetermined FSGS	**–32.092**	(**–51.626, –12.557)**	**.002**	**–23.732**	(**–41.034, –6.430)**	**.008**
(Intercept)	**51.916**	**(43.674, 60.157)**	**<.001**			
Sex						
Male (reference)						
Female	**20.191**	**(6.414, 33.967)**	**.005**	10.683	(–1.073, 22.439)	.074
(Intercept)	**57.865**	**(51.640, 64.090)**	**<.001**			
Age, per decade	**–9.054**	(**–13.248, –4.860)**	**<.001**	**–7.814**	(**–11.658, –3.969)**	**<.001**
(Intercept)	**58.895**	**(51.629, 66.160)**	**<.001**			
UPCR at biopsy, per 1 g/g	**2.531**	**(0.212, 4.851)**	**.033**	–0.958	(–3.304, 1.389)	.418
(Intercept)	**62.161**	**(51.817, 72.506)**	**<.001**			
Glomeruli with FSGS (per 10%)	–1.584	(–6.445, 3.276)	.518	–3.550	(–7.235, 0.135)	.059
(Intercept)	**76.118**	**(68.230, 84.006)**	**<.001**			
Glomeruli with global sclerosis (per 10%)	**–8.473**	(**–11.293, –5.653)**	**<.001**	**–4.810**	(**–7.958, –1.662)**	**.003**
(Intercept)	**89.592**	**(81.091, 98.093)**	**<.001**			
MCCS (per 1)	**–6.992**	(**–8.595, –5.390)**	**<.001**			
**Interaction with time** ^b^						
(Time, in years)	**–2.545**	(**–5.005, –0.084)**	**.043**	0.688	(–2.184, 3.561)	.639
FSGS subgroups						
Primary FSGS (reference)						
Maladaptive FSGS	0.008	(–3.317, 3.333)	.996	–0.870	(–4.333, 2.593)	.622
Genetic FSGS	–2.074	(–6.359, 2.212)	.343	**–4.811**	(**–9.004, –0.619)**	**.025**
Undetermined FSGS	–1.814	(–5.619, 1.991)	.350	–0.096	(–3.394, 3.202)	.954
(Time, in years)	**–4.170**	(**–5.890, –2.450)**	**<.001**			
Sex						
Male (reference)						
Female	2.222	(–0.385, 4.829)	.095	**2.570**	**(0.255, 4.884)**	**.030**
(Time, in years)	**–3.245**	(**–4.590, –1.899)**	**<.001**			
Age, per decade	–0.021	(–0.923, 0.881)	.963	–0.386	(–1.271, 0.499)	.393
(Time, in years)	**–2.716**	(**–4.121, –1.311)**	**<.001**			
UPCR at biopsy, per 1 g/g	–0.260	(–0.662, 0.142)	.204	**–0.538**	(**–0.973, –0.104)**	**.015**
(Time, in years)	–0.513	(–2.285, 1.260)	.570			
Glomeruli with FSGS (per 10%)	**–1.604**	(**–2.391, –0.818)**	**<.001**	**–1.452**	(**–2.183, –0.720)**	**<.001**
(Time, in years)	–1.466	(–3.112, 0.179)	.081			
Glomeruli with global sclerosis (per 10%)	**–0.978**	(**–1.612, –0.345)**	**.002**	–0.569	(–1.298, 0.160)	.126
(Time, in years)	–0.620	(–2.640, 1.400)	.547			
MCCS (per 1)	**–0.619**	(**–1.011, –0.226)**	**.002**			

a Here, estimate (β) estimates the effect of the variable on eGFR at starting timepoint (mL/min/1.73 m^2^), defined as three months after biopsy. “Intercept” can be interpreted as eGFR at this baseline timepoint.

b Here, the estimates (β) are based on interaction effects with time (in years), derived from the models in ^a^. “Time in years” can therefore be interpreted as annual eGFR slope (mL/min/1.73 m^2^ per year).

In this table, uni- and multivariable linear mixed-effects models (including random intercepts and random slopes for time) show the effect of the following clinicopathologic variables on the eGFR intercept (main effect) and annual eGFR slope (interaction with time): FSGS subgroup, sex, age (per decade), proteinuria at biopsy (UPCR, g/g), the percentage of glomeruli affected by FSGS lesions (per 10%), the percentage of glomeruli affected by global sclerosis (per 10%) and MCCS (per 1 point). MCCS was not included in the multivariable analysis, as it is colinear with the percentage of glomeruli affected by FSGS and global sclerosis. The age variable was centered around the median age of 53.8 years in the entire cohort; the proteinuria variable was centered around the median UPCR value of 2.4 g/g in the entire cohort.

Estimates (β) with *P*-value <.05 shown in bold.

Next, a multivariable linear mixed-effects model estimated the effect of the FSGS subgroups, sex, age, proteinuria and percentage of glomeruli affected by FSGS lesions or global sclerosis on baseline eGFR and slope (Table 4). Only genetic FSGS was associated with a significant decrease in eGFR slope [–4.8 mL/min/year compared with primary FSGS, 95% CI (–9.0, –0.6), *P* = .025]. Female sex exhibited no significant effect on baseline eGFR but was associated with a significant increase in slope [+2.6 mL/min/year compared with male patients, 95% CI (0.3, 4.9), *P* = .030]. Increasing patient age at biopsy was associated with a significantly lower baseline eGFR [–7.8 mL/min per decade, 95% CI (–11.7, –4.0), *P* < .001], but showed no significant effect on eGFR slope. Higher proteinuria at kidney biopsy was associated with a small but significant decrease in eGFR slope [–0.5 mL/min/year per 1 g/g, 95% CI (–1.0, –0.1), *P* = .015]. A higher percentage of glomeruli affected by global sclerosis on kidney biopsy was associated with a lower baseline eGFR [–4.8 mL/min per 10% increase, 95% CI (–8.0, –1.7), *P* = .003], while the percentage of glomeruli affected by FSGS lesions was rather associated with decreasing eGFR slope [–1.5 mL/min/year per 10% increase (–2.2, –0.7), *P* < .001].

## DISCUSSION

In this study, all patients with biopsy-proven FSGS from two tertiary referral hospitals in Belgium between 2010 and 2022 were studied. Patients were subclassified according to their underlying etiology, using updated diagnostic criteria [5, 15]. Next, we utilized complementary statistical methods to estimate the effect of the adjudicated FSGS subgroup and clinicopathologic covariates on the risk of kidney failure.

Primary FSGS accounted for 28.1% of all cases, consistent with previous data from the USA [18]. Remarkably, a high proportion of patients were former or active smokers (59.8%), similar to data from a recent German registry study (57.6%) [3]. Patients with undetermined FSGS were often nephrotic (58.3%), exhibited low baseline eGFR (median 35.0 mL/min/1.73 m^2^), featured a high degree of chronicity on biopsy and most often developed kidney failure during follow-up (42.9%). These patients often had several potential secondary causes for FSGS, and the degree of FPE was not always in accordance with the clinical presentation, making the distinction between primary and secondary FSGS very difficult. This is reflected in the inconsistent use of immunosuppressive treatment throughout this subgroup (35.7%). The recent identification of pathogenic circulating autoantibodies in podocytopathies is a promising first step toward novel diagnostic biomarkers in FSGS that would most benefit these patients with FSGS of undetermined etiology [8, 19].

Next, we assessed the effect of the FSGS subgroup on kidney outcomes. Patients with primary FSGS least often developed kidney failure during follow-up (13.0%). After correction for covariates, the FSGS subgroup itself was not associated with kidney failure, while sex, baseline eGFR, proteinuria and the degree of chronicity on kidney biopsy were. In linear mixed-effects models, baseline eGFR was significantly higher in primary FSGS, compared with the other FSGS subgroups, indicating that these patients with overt nephrotic syndrome are biopsied earlier in the disease course. Unexpectedly, the eGFR slopes of the four groups were not significantly different in univariable analysis. After adjusting for age, sex, proteinuria and the degree of segmental and global sclerosis, only genetic FSGS showed a significantly lower eGFR slope. In our study, the estimated annual slope of –2.5 mL/min/year in both primary and maladaptive FSGS was notably better when compared with recent data from the DUPLEX trial, which showed an annual eGFR decline of –5.5 mL/min in a mixed population of patients with FSGS [20]. In contrast, a recent German retrospective study observed better annual eGFR slopes in primary FSGS (–0.96 mL/min/year) and secondary FSGS (–1.88 mL/min/year) [3]. In their study, the baseline timepoint was often several years after diagnosis, while in our study, the baseline timepoint was set at three months after first diagnostic biopsy. Our analysis therefore both considers the early and more chronic phases of kidney disease progression in FSGS.

In our study, female sex was associated with a lower kidney failure rate and higher annual eGFR slope. However, this association was not observed in a recent study from Germany (HR 0.98 in female patients) [3]. Higher proteinuria at biopsy was associated with a significantly higher kidney failure rate, a lower annual eGFR slope and a higher mortality rate, and is therefore associated with overall worse prognosis. A higher percentage of glomeruli affected by segmental (FSGS) or global glomerulosclerosis were both associated with a higher kidney failure rate. Global sclerosis was associated with a significantly lower baseline eGFR (–4.8 mL/min per 10% increase) and indicates limited kidney function reserve. A higher percentage of FSGS lesions rather associated with a significant decrease in annual eGFR slope (–1.5 mL/min/year per 10% increase) and likely indicates more aggressive disease. The median percentage of glomeruli with FSGS lesions was similar across all FSGS subgroups, and a high percentage therefore likely heralds more rapid CKD progression, regardless of the underlying FSGS etiology. Overall, sex, baseline eGFR, proteinuria and the degree of focal and global glomerulosclerosis may play a more important role in estimating the prognosis of patients with FSGS, than merely the FSGS etiology. Nevertheless, the steep downsloping eGFR in genetic FSGS, even after correction for covariates, warrants close follow-up of these patients.

Our study has some limitations. First, our sample size was relatively small, and eGFR follow-up time, with censoring at a median of 36.2 months, was relatively short. Second, although we used strict diagnostic criteria to stratify patients across primary and maladaptive FSGS subtypes [4, 5, 15], we cannot rule out that one or more patients have been misclassified. The undetermined FSGS subgroup, which demonstrated worse kidney outcomes, likely also contained patients with primary FSGS in which a definitive diagnosis could not be made. The remarkably good prognosis of patients with primary FSGS in this study therefore only applies to those that meet the KDIGO diagnostic criteria. Third, we did not include partial and complete remission as surrogate outcomes in our study. In preliminary analyses, we found that the definition of partial and complete remission, as defined by KDIGO guidelines [15], did not perform well in the non-primary FSGS groups. As previously suggested, the definition of partial and complete disease remission should likely be adapted in patients with non-primary FSGS [5].

Our study has several strengths. We carefully phenotyped every FSGS patient, allowing comprehensive analysis of patients with maladaptive, genetic and undetermined FSGS, which have been underrepresented in previous reports. Our analyses accounted for competing events, because merely censoring for such competing events may introduce bias in survival analysis [17]. We estimated eGFR slope, which serves as a surrogate endpoint for kidney failure [21], and included patients at kidney biopsy, allowing us to model the subacute and early chronic kidney function trajectory, which has been lacking in previous studies [3, 20].

In conclusion, patients with primary FSGS were biopsied earlier in the disease course and did not show accelerated kidney function decline, nor higher rates of kidney failure. Male sex, lower baseline eGFR, higher baseline proteinuria and higher degree glomerulosclerosis were associated with adverse outcomes in FSGS, with a higher percentage of segmental sclerosis being associated with more rapid kidney function decline. In approximately one out of six patients, the precise cause of the FSGS lesions remained unknown, leading to various treatments and a poor renal prognosis, necessitating the identification of novel diagnostic biomarkers in FSGS.

## Supplementary Material

sfaf060_Supplemental_File

## Data Availability

The data underlying this article will be shared on reasonable request to the corresponding author.
