# Supplementary material for: Primary FSGS is not associated with worse kidney outcome compared with other FSGS subtypes
Source: Clin Kidney J. 2025 Feb 21;18(4):sfaf060. doi: 10.1093/ckj/sfaf060 (PMC11976529; doi:10.1093/ckj/sfaf060)
Supplement: sfaf060_Supplemental_File [file sfaf060_supplemental_file.docx]

**Supplementary FILE**

**Primary FSGS is not associated with worse kidney outcome compared to other FSGS subtypes**

Dries Deleersnijder^1^, Evert Cleenders^1^, Maarten Coemans^1^, Amélie Dendooven^2,3^, Priyanka Koshy^1,4^, Kathleen Claes^1,5^, Katrien De Vusser^1,5^, Björn K. Meijers^1,5^, Ben Sprangers^6,7^, Steven Van Laecke^8^, Amaryllis H. Van Craenenbroeck^1,5^

^1^ Nephrology and Renal Transplantation Research Group, Department of Microbiology, Immunology and Transplantation, KU Leuven, Leuven, Belgium

^2^ Division of Pathology, University Hospital Ghent, Ghent, Belgium

^3^ Laboratory of Experimental Medicine and Pediatrics, University of Antwerp, Wilrijk, Belgium

^4^ Department of Pathology, University Hospitals Leuven, Leuven, Belgium

^5^ Division of Nephrology, University Hospitals Leuven, Leuven, Belgium

^6^ Department of Nephrology, Ziekenhuis Oost-Limburg, Genk, Belgium

^7^ Department of Immunology and Infection, Biomedical Research Institute, UHasselt, Diepenbeek, Belgium

^8^ Renal Division, Department of Internal Medicine, Ghent University Hospital, Ghent, Belgium

**Corresponding Author:**

Amaryllis Van Craenenbroeck, MD PhD

Nephrology and Renal Transplantation Research Group, KU Leuven

Department of Microbiology, Immunology and Transplantation, KU Leuven.

UZ Herestraat 49, 3000 Leuven, Belgium

Email: amaryllis.vancraenenbroeck@kuleuven.be

ORCID ID: <https://orcid.org/0000-0003-4728-9406>

**Table of Contents**

[Supplementary Methods 3](#_Toc176982072)

[Statistical analysis 3](#_Toc176982073)

[Baseline characteristics 3](#_Toc176982074)

[Linear mixed-effects models for eGFR slope 3](#_Toc176982075)

[Analysis and illustrations 3](#_Toc176982076)

[Supplementary Tables 4](#_Toc176982077)

[Supplementary Table S1: Data missingness 4](#_Toc176982078)

[Supplementary Table S2: Description of the Mayo Clinic Chronicity Score (MCCS) 5](#_Toc176982079)

[Supplementary Table S3: Clinicopathologic characteristics of patients with a diagnosis of undetermined FSGS 6](#_Toc176982080)

[Supplementary Table S4: Gene variants identified in patients with genetic FSGS 8](#_Toc176982081)

[Supplementary Table S5: Follow-up times for kidney failure and death 9](#_Toc176982082)

[Supplementary Table S6: Fine and Gray models for kidney failure 10](#_Toc176982083)

[Supplementary Table S7: Cause-specific Cox proportional hazards models for death before kidney failure 11](#_Toc176982084)

[Supplementary Table S8: Cox proportional hazards models for any death 12](#_Toc176982085)

[Supplementary Figures 13](#_Toc176982086)

[Supplementary Figure S1: Assumption check for multivariable linear mixed-effects model 13](#_Toc176982087)

[REFERENCES 14](#_Toc176982088)

# Supplementary Methods

## Statistical analysis

### Baseline characteristics

All analyses were performed in R (version 4.2.1, R Foundation for Statistical Computing, Vienna, Austria (<https://www.R-project.org/>). Data missingness was low, and, for each analysis, complete data were required for the included variables (**Supplementary Table S1**).

### Linear mixed-effects models for eGFR slope

Estimated GFR values, calculated using the CKD-EPI formula (mL/min/1.73 m^2^), were used to evaluate kidney function over time. Univariable and multivariable linear mixed-effects models were used to estimate the annual eGFR slope, including random intercepts and random slopes. The random intercept allowed for a patient-specific eGFR at baseline, while the random slope allowed for a patient-specific eGFR slope over time. The assumptions of linearity, homoscedasticity and residual normality were not violated (shown for the multivariable model in **Supplementary Figure S1**).

## Analysis and illustrations

Figure 1 was created with GraphPad Prism version 10.2.3 for MacOS (GraphPad Software, [www.graphpad.com](http://www.graphpad.com)). Figure 2 was created with SankeyMATIC ([www.sankeymatic.com](http://www.sankeymatic.com)). Other figures were created in R environment.

# Supplementary Tables

## Supplementary Table S1: Data missingness

|  | **Primary FSGS (N=23)**  N (%) | | **Maladaptive FSGS (N=33)**  N (%) | | **Genetic FSGS (N=12)**  N (%) | | **Undetermined FSGS (N=14)**  N (%) | |
| --- | --- | --- | --- | --- | --- | --- | --- | --- |
| **Demographics and comorbidities** | | | | | | | | |
| Age, at biopsy | 0 | (0.0) | 0 | (0.0) | 0 | (0.0) | 0 | (0.0) |
| Sex | 0 | (0.0) | 0 | (0.0) | 0 | (0.0) | 0 | (0.0) |
| Ethnicity | 0 | (0.0) | 0 | (0.0) | 0 | (0.0) | 0 | (0.0) |
| BMI at biopsy (kg/m^2^) | 1 | (4.3) | 1 | (3.0) | 0 | (0.0) | 0 | (0.0) |
| BMI 1 month after biopsy (kg/m^2^) | 2 | (8.7) | 14 | (42.4) | 4 | (33.3) | 3 | (21.4) |
| OSAS, at biopsy | 0 | (0.0) | 0 | (0.0) | 0 | (0.0) | 0 | (0.0) |
| Hypertension, at biopsy | 0 | (0.0) | 0 | (0.0) | 0 | (0.0) | 0 | (0.0) |
| Current/former smoker, at biopsy | 0 | (0.0) | 0 | (0.0) | 0 | (0.0) | 0 | (0.0) |
| Diabetes mellitus, at biopsy | 0 | (0.0) | 0 | (0.0) | 0 | (0.0) | 0 | (0.0) |
| **Clinical and biochemical characteristics** | | | | | | | | |
| RAAS inhibitor use, at biopsy | 0 | (0.0) | 0 | (0.0) | 0 | (0.0) | 0 | (0.0) |
| Nephrotic syndrome, at biopsy | 0 | (0.0) | 0 | (0.0) | 1 | (8.3) | 2 | (14.3) |
| Edema, at biopsy | 0 | (0.0) | 2 | (6.1) | 0 | (0.0) | 0 | (0.0) |
| Hematuria, at biopsy | 1 | (4.3) | 2 | (6.1) | 1 | (8.3) | 1 | (7.1) |
| UPCR, highest^a^ | 0 | (0.0) | 0 | (0.0) | 0 | (0.0) | 0 | (0.0) |
| UPCR, at biopsy | 0 | (0.0) | 3 | (9.1) | 3 | (25.0) | 2 | (14.3) |
| sAlb, at biopsy (g/L) | 0 | (0.0) | 0 | (0.0) | 2 | (16.7) | 0 | (0.0) |
| sCr, at biopsy (mg/dL) | 0 | (0.0) | 0 | (0.0) | 0 | (0.0) | 0 | (0.0) |
| eGFR, at biopsy (mL/min/1.73m^2^) | 0 | (0.0) | 0 | (0.0) | 0 | (0.0) | 0 | (0.0) |
| **Histopathologic features** | | | | | | | | |
| Percentage of glomeruli with FSGS lesions on LM | 0 | (0.0) | 1 | (3.0) | 0 | (0.0) | 0 | (0.0) |
| Percentage of glomeruli with global sclerosis on LM | 0 | (0.0) | 0 | (0.0) | 0 | (0.0) | 0 | (0.0) |
| GS | 0 | (0.0) | 0 | (0.0) | 0 | (0.0) | 0 | (0.0) |
| IF | 1 | (4.3) | 0 | (0.0) | 1 | (8.3) | 0 | (0.0) |
| TA | 1 | (4.3) | 0 | (0.0) | 1 | (8.3) | 0 | (0.0) |
| CV | 1 | (4.3) | 0 | (0.0) | 1 | (8.3) | 0 | (0.0) |
| MCCS | 1 | (4.3) | 0 | (0.0) | 1 | (8.3) | 0 | (0.0) |
| EM performed | 0 | (0.0) | 0 | (0.0) | 0 | (0.0) | 0 | (0.0) |
| **Genetics** | | | | | | | | |
| Genetics performed | 0 | (0.0) | 0 | (0.0) | 0 | (0.0) | 0 | (0.0) |

This table shows the number of missing data values (N and %).^a^ highest proteinuria in the time interval of 3 months up until biopsy.

BMI: body mass index, CV: arteriosclerosis (score 0-1, part of total MCCS), eGFR: estimated glomerular filtration rate, EM: electron microscopy, GS: glomerulosclerosis (score 0-3, part of total MCCS), IF: interstitial fibrosis (score 0-3, part of total MCCS), LM: light microscopy, MCCS: Mayo Clinic Chronicity Score, OSAS: obstructive sleep apnea syndrome, RAAS: renin-angiotensin-aldosterone system, sAlb: serum albumin, sCr: serum creatinine, TA: tubular atrophy (score 0-3, part of total MCCS), UPCR: urine protein-to-creatinine ratio

## Supplementary Table S2: Description of the Mayo Clinic Chronicity Score (MCCS)

| **Tissue compartment** | **Score** | | | |
| --- | --- | --- | --- | --- |
|  | **0** | **1** | **2** | **3** |
| Glomerulosclerosis  (GS score) | <10% | 10-25% | 26-50% | >50% |
| Interstitial fibrosis  (IF score) | <10% | 10-25% | 26-50% | >50% |
| Tubular atrophy  (TA score) | <10% | 10-25% | 26-50% | >50% |
| Arteriosclerosis  (CV score) | Intimal thickness < thickness of media | Intimal thickness ≧ thickness of media |  |  |

Adapted from Sethi *et al*.^1^ GS score includes the percentage of glomeruli with global and segmental sclerosis and ischemic glomeruli; IF and TA score includes the percentage of kidney cortex involved by interstitial fibrosis and tubular atrophy, respectively; CV score includes the severity of arteriosclerosis determined by the extent of thickening of the intima.

## Supplementary Table S3: Clinicopathologic characteristics of patients with a diagnosis of undetermined FSGS

| **ID** | **Sex** | **Age** | **BMI** | **Secondary causes** | **Edema** | **Smoker** | **UPCR** | **sAlb** | **eGFR** | **NS** | **IF/IHC** | **(%) FSGS** | **(%) GS** | **MCCS** | **Columbia** | **FPE** | **Treatment** | **KF** | **Death** |
| --- | --- | --- | --- | --- | --- | --- | --- | --- | --- | --- | --- | --- | --- | --- | --- | --- | --- | --- | --- |
| **1** | F | 70.6 | 27.2 | None | Yes | Non-smoker | 2.0 | 27.6 | 45 | No | Negative | 16.7% | 16.7% | 4 | NA | NA | CS mono | Yes, 39.3 mo | No |
|  | **Description**: Suggestive of primary FSGS, but not nephrotic at biopsy. One year later relapse with full-blown nephrotic syndrome, treated with CS. | | | | | | | | | | | | | | | | | | |
| **2** | M | 48.3 | 33.7 | Obesity | Yes | Smoker | 4.2 | 32.3 | 97 | Yes | Negative | 5.9% | 5.9% | 1 | NA | NA | CS + CNI | No | No |
|  | **Description**: Suggestive of maladaptive FSGS, but incomplete response to RAASi. CS + CNI initiated, complete remission after many years. | | | | | | | | | | | | | | | | | | |
| **3** | F | 42.5 | 26.9 | None | Yes | Non-smoker | 3.0 | 36.4 | 32 | No | Negative | 12.0% | 4.0% | 3 | NA | ‘diffuse FPE' | CS mono | No | No |
|  | **Description**: Compatible with primary FSGS, but subnephrotic proteinuria. Complete remission with CS. | | | | | | | | | | | | | | | | | | |
| **4** | M | 55.7 | 37.3 | Vasc, obesity, paraneoplastic (?) | Yes | Ex-smoker | 10.5 | 18.1 | 19 | Yes | Negative | 5.9% | 11.8% | 2 | NA | NA | CS mono | No | Yes, 33.8 mo |
|  | **Description**: Suggestive of 'paraneoplastic' primary FSGS, diagnosed 1 month after diagnosis of metastasized renal cell carcinoma. Remission with CS. Disease recurrence upon tumor progression. | | | | | | | | | | | | | | | | | | |
| **5** | F | 61.4 | 31.2 | DM, Vasc, obesity | No | Smoker | 3.6 | 28.2 | 86 | NA | C3 (trace) | 33.3% | 33.3% | 8 | NOS type | NA | None | Yes, 67.7 mo | No |
|  | **Description**: Likely maladaptive cause, but past medical history of sarcoidosis and transient nephrotic syndrome. | | | | | | | | | | | | | | | | | | |
| **6** | F | 55.3 | 29.1 | Vasc | No | Ex-smoker | 5.6 | 32.2 | 45 | Yes | IgM and C3 (trace) | 15.4% | 15.4% | 6 | Collapsing type | NA | None | No | No |
|  | **Description**: Difficult DDx: initially nephrotic and collapsing FSGS on kidney biopsy, but spontaneously subnephrotic and normal kidney function 8 years later without any immunosuppressive treatment. | | | | | | | | | | | | | | | | | | |
| **7** | F | 74.9 | 24.9 | DM, Vasc, NSAID | Yes | Ex-smoker | 20.5 | 27.3 | 37 | Yes | Negative | 11.8% | 64.7% | 9 | Collapsing type | NA | None | Yes, 17.2 mo | Yes, 33.3 mo |
|  | **Description**: Difficult DDx: nephrotic at diagnosis, but also many possible secondary causes (DM, Vasc, NSAID). Reduction of proteinuria with supportive care. | | | | | | | | | | | | | | | | | | |
| **8** | M | 75.7 | 26.8 | None | Yes | Non-smoker | 5.0 | 41.8 | 28 | No | C3 (+) | 12.5% | 70.8% | 10 | 1 perihilar, 2 tip lesions | ≥80% | None | Yes, 5.0 mo | No |
|  | **Description**: Discordance between FPE ≥ 80%, but absence of nephrotic syndrome. Supportive treatment. | | | | | | | | | | | | | | | | | | |
| **9** | M | 42.3 | 37.3 | OSAS, obesity | No | Smoker | 5.8 | 33.2 | 26 | NA | Negative | 29.4% | 58.8% | 10 | perihilar type | NA | None | No | No |
|  | **Description**: Difficult DDx: maladaptive causes present, transient nephrotic syndrome. Lowering of proteinuria with supportive care but kidney disease progression to kidney failure. | | | | | | | | | | | | | | | | | | |
| **10** | F | 21.1 | 26.9 | None | Yes | Non-smoker | 13.3 | 20.5 | 27 | Yes | Negative | 0.0% | 0.0% | 0 | NA | <80% | CS mono,  CS + RTX,  CS + CNI | No | No |
|  | **Description**: Discordance between FPE < 80% and full-blown nephrotic syndrome at biopsy (prior to treatment). | | | | | | | | | | | | | | | | | | |
| **11** | M | 55.3 | 25.7 | None | No | Ex-smoker | 3.3 | 35.7 | 77 | No | Negative | 5.9% | 11.8% | 1 | NOS type | ≥80% | None | No | No |
|  | **Description**: Discordance between FPE ≥ 80% but normal serum albumin at biopsy. No response to sparsentan. Likely primary FSGS, despite absence of nephrotic syndrome. | | | | | | | | | | | | | | | | | | |
| **12** | F | 58.0 | 36.5 | Heroin, DM, HBV, HCV, sofosbuvir, obesity | No | Smoker | 6.9 | 27.3 | 77 | Yes | IgM and C3 (trace) | 33.3% | 33.3% | 7 | NOS type | <80% | None | No | Yes, 30.8 mo |
|  | **Description**: Difficult DDx: many secondary causes (heroin abuse, DM, HBV, HCV, sofosbuvir, obesity). | | | | | | | | | | | | | | | | | | |
| **13** | M | 33.3 | 25.7 | None | No | Non-smoker | 3.8 | 32.5 | 33 | Yes | Negative | 20.0% | 70.0% | 10 | perihilar type | <80% | None | Yes, 12.9 mo | No |
|  | **Description**: Discordance between FPE < 80%, but nephrotic syndrome at biopsy. Suggestive of maladaptive FSGS, but no clear maladaptive etiology. | | | | | | | | | | | | | | | | | | |
| **14** | M | 55.3 | 25.8 | HIV, antiretroviral therapy | No | Non-smoker | 3.2 | 39.0 | 32 | No | IgM and C3 (+) | 62.5% | 25.0% | 7 | NOS | ≥80% | None | Yes, 22.4 mo | No |
|  | **Description**: Difficult DDx: FPE ≥ 80%, but no nephrotic syndrome at biopsy. Antiretroviral therapy as possible cause. HIV viral load well controlled. | | | | | | | | | | | | | | | | | | |

(%) FSGS: ratio of glomeruli with FSGS lesion(s) to total glomeruli on light microscopy (%), (%) GS: ratio of glomeruli with global sclerosis to total glomeruli on light microscopy (%), age: age at biopsy (yrs.), BMI: body mass index (kg/m2), Columbia: Columbia classification of FSGS lesions, CS: corticosteroids, CNI: calcineurin inhibitor, Death: death (time to event in months, from kidney biopsy), DM: diabetes mellitus, DDx: differential diagnosis, Edema: presence of edema at biopsy, eGFR: estimated GFR at biopsy (mL/min/1.73m^2^), F: female, FPE: degree of podocyte foot process effacement on electron microscopy, HBV: hepatitis B virus, HCV: hepatitis C virus, ID: patient ID, IF/IHC: result from glomerular immunofluorescence or immunohistochemistry stains, KF: kidney failure (time to event in months, from kidney biopsy), MCCS: Mayo Clinic Chronicity Score, M: male, mo: months, mono: monotherapy, NOS: not otherwise specified, NS: presence of nephrotic syndrome at kidney biopsy, NSAID: non-steroidal anti-inflammatory drugs, OSAS: obstructive sleep apnea, RTX: rituximab, sAlb: serum albumin at biopsy (g/L), Smoker: smoking status at kidney biopsy, UPCR: highest proteinuria (urine protein-creatinine ratio) in the time interval of 3 months up until biopsy, Vasc: past medical history of macrovascular disease at kidney biopsy (coronary heart disease and/or cerebrovascular disease and/or peripheral artery disease).

## Supplementary Table S4: Gene variants identified in patients with genetic FSGS

| **Patient** | **Gene affected** | **Inheritance** | **cDNA** | **Amino acid** | **Zygosity** | **Variant class** |
| --- | --- | --- | --- | --- | --- | --- |
| 1 | *COL4A3* (NM_000091.4) | AD/AR | c.2135G>A | p.Gly712Asp | Heterozygous | Pathogenic |
| 2 | *COL4A3* (NM_000091.4) | AD/AR | c.2083G>A | p.Gly695Arg | Heterozygous | Likely pathogenic |
| 3 | *COL4A3* (NM_000091.4) | AD/AR | c.2329G>T | p.Gly777Cys | Heterozygous | Pathogenic |
| 4 | *INF2*  (NM_0022489.3) | AD | c.641G>A | p.Arg214His | Heterozygous | Pathogenic |
| 5 | *NPHS2*  (NM_014625.3) | AR | c.855_856del | p.Arg286Thrfs*17 | Heterozygous | Likely pathogenic |
| 6 | *COL4A5* (NM_000495.4) | X-linked | c.4539T>G | p.Phe1513Leu | Heterozygous | Likely pathogenic |
| 7 | *COL4A3* (NM_000091.4) | AD/AR | c.279+1G>T | splice mutation | Heterozygous | Likely pathogenic |
| 8 | *COL4A5* (NM_000495.4) | X-linked | c.4819G>A | p.Ala1607Thr | Heterozygous | Likely pathogenic |
| 9 | *COL4A3* (NM_000091.4) | AD/AR | c.2329G>T | p.Gly777Cys | Heterozygous | Pathogenic |
| 10 | *COL4A3* (NM_000091.4) | AD/AR | c.2329G>T | p.Gly777Cys | Heterozygous | Pathogenic |
| 11 | *LMX1B*  (NM_002316.3) | AD | c.337del | p.Ala113Profs*16 | Heterozygous | Likely pathogenic |
| 12 | *INF2*  (NM_0022489.3) | AD | c.652C>T | p.Arg218Trp | Heterozygous | Likely pathogenic |

AD: autosomal dominant; AR: autosomal recessive; cDNA: coding DNA sequence

## Supplementary Table S5: Follow-up times for kidney failure and death

|  | **Number of**  **patients** | **Follow-up time (months)**  **Median (IQR)** | |
| --- | --- | --- | --- |
| **Kidney failure** |  |  |  |
| Time to kidney failure | 22 | 34.5 | (18.3-38.9) |
| Time to censoring on death or last eGFR measurement | 60 | 36.2 | (23.0-68.5) |
| **Death**^a^ |  |  |  |
| Time to death | 10 | 29.1 | (13.0-32.7) |
| Time to censoring on last medical contact | 72 | 52.4 | (32.9-89.6) |

^a^ Causes of death include malignancy (N = 3), unknown (N = 3), aspiration pneumonia (N = 1), endocarditis (N = 1), hypercapnic respiratory failure (N = 1) and intracranial hemorrhage (N = 1).

## Supplementary Table S6: Fine and Gray models for kidney failure

|  | **Univariable** | | |  | **Multivariable** | | |
| --- | --- | --- | --- | --- | --- | --- | --- |
| **Parameter** | **sHR** | **(95% CI)** | ***P*-value** |  | **sHR** | **(95% CI)** | ***P*-value** |
| FSGS subgroups |  |  |  |  |  |  |  |
| Primary FSGS (reference) |  |  |  |  |  |  |  |
| Maladaptive FSGS | 1.966 | (0.536, 7.207) | 0.308 |  | 1.309 | (0.042, 40.759) | 0.878 |
| Genetic FSGS | 3.307 | (0.829, 13.186) | 0.090 |  | 3.293 | (0.120, 90.199) | 0.480 |
| Undetermined FSGS | 2.996 | (0.755, 11.884) | 0.119 |  | 1.575 | (0.378, 6.562) | 0.533 |
| Sex |  |  |  |  |  |  |  |
| Male (reference) |  |  |  |  |  |  |  |
| Female | 0.446 | (0.175, 1.136) | 0.091 |  | 0.329 | (0.100, 1.086) | 0.068 |
| Age, per decade | 1.243 | (0.954, 1.620) | 0.107 |  | 1.253 | (0.808, 1.943) | 0.314 |
| eGFR at biopsy, per 10 mL/min/1.73 m^2^ | **0.688** | **(0.552, 0.857)** | **0.001** |  | **0.744** | **(0.594, 0.932)** | **0.010** |
| UPCR at biopsy, per 1 g/g | 1.070 | (0.938, 1.221) | 0.314 |  | 1.201 | (0.778, 1.854) | 0.409 |
| Glomeruli with FSGS (per 10%) | **1.245** | **(1.005, 1.542)** | **0.045** |  | **1.777** | **(1.401, 2.255)** | **<0.001** |
| Glomeruli with global sclerosis (per 10%) | **1.699** | **(1.401, 2.061)** | **<0.001** |  | **2.050** | **(1.390, 3.024)** | **<0.001** |
| MCCS (per 1) | **1.637** | **(1.369, 1.958)** | **<0.001** |  |  |  |  |

Univariable and multivariable (competing risks) Fine and Gray models estimating the effect of the following clinicopathologic variables on the risk of kidney failure: FSGS subgroup, sex, age (per decade), eGFR at biopsy (per 10 mL/min/1.73 m2), proteinuria at biopsy (UPCR, g/g), the percentage of glomeruli affected by FSGS lesions (per 10%), the percentage of glomeruli affected by global sclerosis (per 10%) and MCCS (per 1 point). Models account for the competing event ‘death before kidney failure’. CI: confidence interval, sHR: subdistribution hazard ratio.

## Supplementary Table S7: Cause-specific Cox proportional hazards models for death before kidney failure

|  | **Univariable** | | |  | **Multivariable** | | |
| --- | --- | --- | --- | --- | --- | --- | --- |
| **Parameter** | **HR** | **(95% CI)** | ***P*-value** |  | **HR** | **(95% CI)** | ***P*-value** |
| FSGS subgroups |  |  |  |  |  |  |  |
| Primary FSGS (reference) |  |  |  |  |  |  |  |
| Maladaptive FSGS | 3.653 | (0.427, 31.289) | 0.237 |  | 28.365 | (0.233, 3457.500) | 0.172 |
| Genetic FSGS | 0.000 | (0.000, Inf) | 0.998 |  | 0.000 | (0.000, Inf) | 0.999 |
| Undetermined FSGS | 3.076 | (0.278, 33.984) | 0.359 |  | 8.787 | (0.488, 158.126) | 0.141 |
| Sex |  |  |  |  |  |  |  |
| Male (reference) |  |  |  |  |  |  |  |
| Female | 0.155 | (0.019, 1.286) | 0.084 |  | 0.242 | (0.019, 3.033) | 0.272 |
| Age, per decade | **2.653** | **(1.356, 5.192)** | **0.004** |  | **4.240** | **(1.239, 14.514)** | **0.021** |
| eGFR at biopsy, per 10 mL/min/1.73 m^2^ | **0.635** | **(0.417, 0.967)** | **0.034** |  | 1.173 | (0.626, 2.200) | 0.618 |
| UPCR at biopsy, per 1 g/g | 1.116 | (0.924, 1.348) | 0.253 |  | 1.724 | (0.981, 3.029) | 0.058 |
| Glomeruli with FSGS (per 10%) | 0.766 | (0.400, 1.468) | 0.422 |  |  |  |  |
| Glomeruli with global sclerosis (per 10%) | 0.978 | (0.655, 1.461) | 0.915 |  |  |  |  |
| MCCS (per 1) | 1.151 | (0.913, 1.452) | 0.235 |  |  |  |  |

Univariable and multivariable Cox proportional hazards models estimating the effect of the following clinicopathologic variables on the cause-specific hazard of death before kidney failure: FSGS subgroup, sex, age (per decade), eGFR at biopsy (per 10 mL/min/1.73 m^2^), proteinuria at biopsy (UPCR, g/g), the percentage of glomeruli affected by FSGS lesions (per 10%), the percentage of glomeruli affected by global sclerosis (per 10%) and MCCS (per 1 point). Censoring was applied for the competing event kidney failure. CI: confidence interval, HR: (cause-specific) hazard ratio.

## Supplementary Table S8: Cox proportional hazards models for any death

|  | **Univariable** | | |  | **Multivariable** | | |
| --- | --- | --- | --- | --- | --- | --- | --- |
| **Parameter** | **HR** | **(95% CI)** | ***P*-value** |  | **HR** | **(95% CI)** | ***P*-value** |
| FSGS subgroups |  |  |  |  |  |  |  |
| Primary FSGS (reference) |  |  |  |  |  |  |  |
| Maladaptive FSGS | 1.585 | (0.307, 8.188) | 0.583 |  | 31.117 | (0.454, 2130.743) | 0.111 |
| Genetic FSGS | 0.000 | (0.000, Inf) | 0.998 |  | 0.000 | (0.000, Inf) | 0.999 |
| Undetermined FSGS | 1.868 | (0.311, 11.219) | 0.494 |  | 9.258 | (0.410, 208.986) | 0.162 |
| Sex |  |  |  |  |  |  |  |
| Male (reference) |  |  |  |  |  |  |  |
| Female | 0.552 | (0.142, 2.142) | 0.390 |  | 0.220 | (0.022, 2.164) | 0.194 |
| Age, per decade | **2.658** | **(1.439, 4.908)** | **0.002** |  | **3.582** | **(1.358, 9.445)** | **0.010** |
| eGFR at biopsy, per 10 mL/min/1.73 m^2^ | **0.696** | **(0.498, 0.973)** | **0.034** |  | 1.254 | (0.707, 2.224) | 0.440 |
| UPCR at biopsy, per 1 g/g | **1.214** | **(1.075, 1.370)** | **0.002** |  | **1.629** | **(1.153, 2.300)** | **0.006** |
| Glomeruli with FSGS (per 10%) | 0.651 | (0.333, 1.272) | 0.209 |  |  |  |  |
| Glomeruli with global sclerosis (per 10%) | 0.981 | (0.724, 1.331) | 0.904 |  |  |  |  |
| MCCS (per 1) | 1.098 | (0.904, 1.333) | 0.346 |  |  |  |  |

Univariable and multivariable Cox proportional hazards models estimating the effect of the following clinicopathologic variables on the hazard of any death: FSGS subgroup, sex, age (per decade), eGFR at biopsy (per 10 mL/min/1.73 m^2^), proteinuria at biopsy (UPCR, g/g), the percentage of glomeruli affected by FSGS lesions (per 10%), the percentage of glomeruli affected by global sclerosis (per 10%) and MCCS (per 1 point). No competing events are present. CI: confidence interval, HR: (cause-specific) hazard ratio.

# Supplementary Figures

**
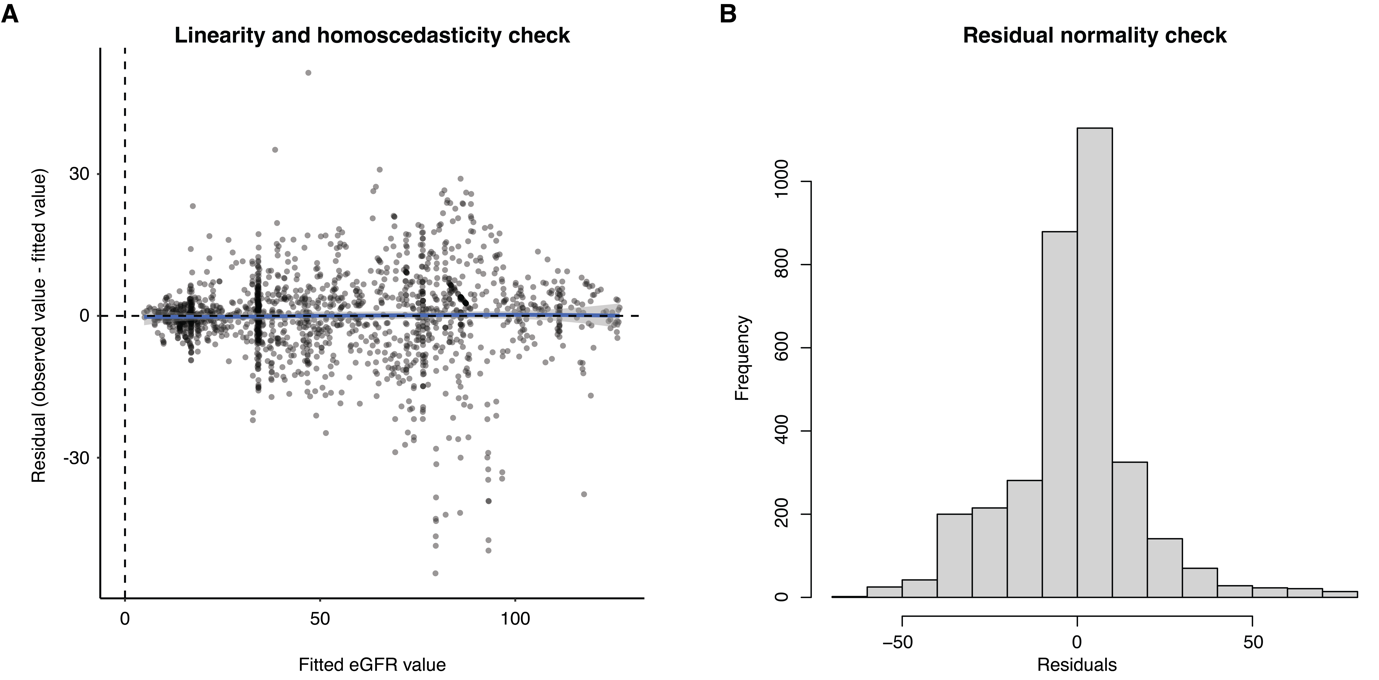
**

## Supplementary Figure S1: Assumption check for multivariable linear mixed-effects model

(**A**) Residual plot with smooth locally estimated scatterplot smoothing (LOESS) curve of the multivariable regression model. The x-axis represents the fitted eGFR value according to the multivariable linear mixed-effects model. The y-axis represents the residuals (observed value – fitted value). The blue line is a smooth LOESS curve which describes the trend in the residuals without specification of a global function (grey area represents confidence interval around curve). The horizontal dashed line at y = 0 (*i.e*., no residual and perfect fit to regression line) is always within the LOESS curve confidence interval, and the condition of linearity is therefore not violated. Overall, the variance of the residuals is constant, and the condition of homoscedasticity is also not violated. (**B**) Histogram of residuals in the multivariable regression model, which shows normal distribution. The condition of residual normality is therefore not violated.

# REFERENCES

1. Sethi S, D'Agati VD, Nast CC, et al. A proposal for standardized grading of chronic changes in native kidney biopsy specimens. Kidney Int. 2017;91(4):787-9.
